# Prévalence de la coronaropathie chez les survivants d’accident vasculaire cérébral à Parakou (Bénin) en 2019

**DOI:** 10.11604/pamj.2021.38.179.22609

**Published:** 2021-02-17

**Authors:** Yessito Corine Nadège Houehanou, Agbetou Mendinatou, Kossi Oyéné, Kamal Zacari, Fiath Yemadjro, Thierry Adoukonou

**Affiliations:** 1 Ecole Nationale de formation des Techniciens Supérieurs en Santé Publique et Surveillance, Epidémiologique (ENATSE), Université de Parakou, Parakou, Bénin,; 2 Service de Neurologie, Centre Hospitalier Universitaire Départemental Borgou-Alibori, Parakou, Bénin,; 3 Unité d'Enseignement et de Recherche de Neurologie, Faculté de Médecine, Université de Parakou, Bénin

**Keywords:** Coronaropathie, accident vasculaire cérébral, Bénin, Coronary artery disease, stroke, Benin

## Abstract

**Introduction:**

la coronaropathie serait fréquente chez les survivants d´accident vasculaire cérébral (AVC). L´objectif de l´étude était de déterminer la prévalence de la coronaropathie chez les survivants d´AVC suivis au Centre Hospitalier Universitaire Départemental Borgou-Alibori (CHUD-B/A).

**Méthodes:**

il s´est agi d´une étude transversale menée du 1^er^ mars au 31 août 2019. Elle a inclus tous les patients victimes d´AVC, d´âge ≥ 18 ans, reçus dans l´unité de neurologie du CHUD-B/A entre janvier 2012 et juillet 2019, ayant survécu à la phase aiguë, et chez qui le consentement éclairé écrit ou celui d´un tuteur (patients sévèrement handicapés) a été obtenu. Un entretien individuel, des mesures anthropométriques, un examen physique, un électrocardiogramme et une revue du dossier médical ont été effectués. La présence de coronaropathie a été définie par un antécédent documenté, des anomalies ST/T et/ou Q évocatrices (critères de Minnesota) ou un questionnaire de dépistage de l´angine de poitrine positif. Les facteurs associés à la coronaropathie ont été recherchés par une régression logistique.

**Résultats:**

un total de 101 patients était inclus avec un âge moyen de 57,2±10,5 ans. Le délai médian depuis la survenue de l´AVC était de 11 mois [2,0-23,5]. Les AVC ischémiques prédominaient (57,4%). La prévalence de la coronaropathie était estimée à 49,5%. Les facteurs associés à la coronaropathie étaient la non-scolarisation (p=0,036), l´obésité (p=0,036) et le tabagisme ancien (p=0,044).

**Conclusion:**

cette étude montre une fréquence élevée de coronaropathie chez les survivants d´AVC. Il est important de dépister la coronaropathie dans cette cible afin d´optimiser la prise en charge.

## Abstract (en)

**Introduction:**

coronary artery disease seem common in stroke patients. We aimed to determine the prevalence of coronary artery disease in stroke survivors followed at the Borgou-Alibori Departmental University Hospital (CHUD/B-A).

**Methods:**

this was a cross-sectional study, conducted from March 1 to August 31, 2019. It included all stroke patients, aged ≥ 18 years, received in the neurology unit of CHUD-B/A between January 2012 and July 2019, having survived the acute phase, and from whom written informed consent or that of a guardian (severely disabled patients) has been obtained. An individual interview, anthropometric measurements, a physical examination, an electrocardiogram record and a review of the medical record were performed. The coronary artery disease was defined by a documented history, suggestive ST/T and/or Q abnormalities (Minnesota criteria) or a positive angina screening questionnaire. The factors associated with coronary artery disease were investigated by logistic regression.

**Results:**

a total of 101 patients were included with an average age of 57.2 ± 10.5 years. The median time since stroke onset was 11 months (interquartile range: [2.0-23.5]). Ischemic stroke predominated (57.4%). The prevalence of coronary artery disease was estimated at 49.5%. The factors associated were lack of school education (p=0.036), obesity (0.036) and former tobacco use (p=0.044).

**Conclusion:**

this study shows a high frequency of coronary artery disease in stroke patients. It is important to detect coronary artery disease in this target population in order to improve the management.

## Introduction

Les maladies cardiovasculaires représentent la première cause de mortalité dans le monde et surtout les accidents vasculaires cérébraux (AVC) et les cardiopathies ischémiques (coronaropathies) [1]. Il n´est pas rare de retrouver une coexistence entre ces deux pathologies. L´athérosclérose est la principale cause des coronaropathies et des AVC ischémiques; il a été identifié des facteurs de risque quasi-identiques pour ces deux pathologies. Une fréquence plus élevée de coronaropathie chez les patients victimes d´AVC qu´en population générale est observée selon la littérature [2,3]. La coronaropathie reste peu recherchée chez les individus après un AVC surtout dans les pays à faible revenu comme le Bénin du fait de l´indisponibilité du plateau technique adéquat. Cependant l´électrocardiogramme pourrait aider à fortement suspecter les coronaropathies asymptomatiques. Différentes anomalies systématisées sont retrouvées sur l´électrocardiogramme de repos (anomalies ST/T, anomalies de l´onde Q) pouvant faire évoquer le diagnostic de coronaropathie [4,5]. Dans plusieurs études portant sur les AVC, les anomalies ST/T étaient fréquentes sur l´électrocardiogramme de repos et associées à un mauvais pronostic [6-8].

Dans le souci d´évaluer l´importance de la coronaropathie chez les sujets victimes d´AVC, une étude avait été menée au Centre Hospitalier Universitaire Départemental Borgou-Alibori (CHUD-B/A) en 2013. Cette étude a permis d´estimer une prévalence de coronaropathie de 47,4% chez les patients en phase aiguë d´AVC [9]. Or la prévalence s´élèverait avec une augmentation du délai de l´AVC en rapport avec l´accroissement des facteurs de risque dans le temps et leur mauvais contrôle. Ainsi elle peut être plus importante après la phase aiguë chez les survivants d´AVC. Les données sur la coronaropathie après une phase aiguë d´AVC sont rares en Afrique et au Bénin en particulier. L´objectif de cette étude était de déterminer la prévalence de la coronaropathie chez les survivants d´AVC suivis au Centre Hospitalier Universitaire Départemental Borgou-Alibori.

## Méthodes

**Cadre et nature de l´étude:** cette étude s´est déroulée dans l´unité de neurologie du CHUD-B/A, du 1^er^ mars au 31 août 2019. Il s´est agi d´une étude transversale, à visée descriptive et analytique. Elle a inclus tous les patients victimes d´AVC âgés d´au moins 18 ans, reçus dans l´unité de neurologie du CHUD-B/A entre le 1^er^ janvier 2012 et le 31 juillet 2019, ayant survécu à la phase aiguë, et chez qui le consentement éclairé écrit ou celui d´un tuteur (pour les patients sévèrement handicapés) a été obtenu. Nous avons exclu les patients dont les tracés électrocardiographiques étaient mal enregistrés ou non interprétables. Le diagnostic d´AVC était basé sur les critères de l´Organisation mondiale de la Santé (OMS) avec ou sans imagerie cérébrale [10].

**Collecte des données:** une entrevue face à face a eu lieu avec chaque patient inclus, suivie: des mesures de paramètres anthropométriques (poids, taille), de la pression artérielle et d´une glycémie capillaire à jeun; de la réalisation d´un examen physique et d´un électrocardiogramme à 12 dérivations. Les électrocardiogrammes ont été réalisés chez les patients torses nus, au repos, allongés sur une table d´examen dans une salle à température ambiante, (20-25°C) grâce à un électrocardiographe (cardiotouch 3000, BIONET, Corée du Sud). Les données anamnestiques ont été recueillies soit dans les dossiers médicaux, soit dans les carnets de sortie. Les données ont été collectées grâce à une fiche de recueil standardisée électronique intégrée au smartphone. L´outil de collecte avait été pré-testé et réajusté avant le début de la collecte de données. Les patients éligibles n´ayant pas consulté ou été hospitalisés durant la période d´étude ont été joints par contact téléphonique; un rendez-vous a été donné à ceux qui ont accepté de participer à l´enquête. Les patients hospitalisés ont été vus dans leur lit d´hospitalisation. L´interprétation des tracés a été faite sur la base des critères du Minnesota par un cardiologue en aveugle des données cliniques.

**Variables:** la variable dépendante était la présence de coronaropathie. Elle était définie par: (1) la présence d´anomalies majeures ST/T ou onde Q (aspect de séquelles de nécrose myocardique) à l´électrocardiogramme de repos (codes Minnesota: 1-1-1; 1-2-3; 4-1; 9-2; 5-1; 5-2; 5-4) [11]; ou (2) un antécédent confirmé de coronaropathie (angor stable, angor instable ou infarctus du myocarde) sur la base du dossier médical ou du compte rendu d´un médecin cardiologue; ou (3) le dépistage d´angor stable à partir du questionnaire de l´OMS [12]. Les variables indépendantes étaient: les caractéristiques sociodémographiques (âge, sexe, niveau d´instruction), les antécédents personnels et familiaux de maladie cardiovasculaire, l´histoire de l´AVC, les facteurs de risque cardiovasculaire. Concernant les facteurs de risque cardiovasculaire, le diabète a été retenu en présence de prise de traitement par insuline ou d´un antidiabétique oral ou d´une glycémie capillaire à jeun ≥ 1,26 g/l le jour de la visite médicale. L'hypertension artérielle a été définie par la prise actuelle d´antihypertenseurs et/ou une pression systolique ≥ 140 mm Hg et/ou une pression diastolique ≥ 90 mm Hg mesurée le jour de la visite médicale (moyenne de 3 mesures consécutives espacées d´au moins 5 minutes). La dyslipidémie a été retenue chez toute personne présentant l´une des anomalies suivantes: hypercholestérolémie totale > 2g/l; hypertriglycéridémie > 1,5 g/l; LDL-Cholestérol élevé > 1g/l; HDL-Cholestérol bas < 0,4 g/l chez l´homme et < 0,5g/l chez la femme. L´obésité a été définie par un indice de masse corporelle (IMC) ≥ 30 kg/m^2^. Le tabagisme a été défini par la consommation de produit fabriqué à base de feuilles de tabac. Les normes de la consommation de fruits et légumes choisies étaient celles instaurées par l´OMS (au moins 5 portions de fruits et légumes par jour). Pour la pratique d´activité physique, le seuil de 150 minutes minimum d´activité modérée ou équivalent par semaine a été considéré [13]. Le degré du handicap a été évalué à partir du score de RANKIN modifié. Le handicap a été considéré comme léger lorsque le score est ≤ 1; modéré lorsque le RANKIN est compris entre 2 et 3; et sévère pour un RANKIN ≥ 4.

**Analyse des données:** les données recueillies ont été traitées grâce au logiciel Epi Info 7.2.2.6 (CDC, Atlanta, USA, 2018). Les variables qualitatives ont été exprimées en proportion tandis que les variables quantitatives en moyenne ± écart-type en cas de distribution normale. Pour étudier l´association entre la présence de coronaropathie et chacune des principales variables indépendantes, une régression logistique uni-variée a été faite; les odds ratios bruts et leurs intervalles de confiance à 95% (IC à 95%) ont été estimés. Toutes les variables ayant un p<0,2 ont ensuite été introduites simultanément dans un modèle de régression logistique multivariée, selon une méthode pas à pas descendante.

**Considérations éthiques:** le secret médical et les droits des participants ont été respectés tout au long de l'étude. En outre, les patients enquêtés ont été rassurés quant à l´anonymat et à la confidentialité des informations collectées. Le protocole a reçu l´approbation du Comité local d´éthique de la recherche biomédicale de l´Université de Parakou: autorisation n˚ 0284/CLERB-UP/P/R/SA. Les patients ayant une coronaropathie ont été orientés vers un cardiologue pour l´optimisation de leur prise en charge.

## Résultats

**Caractéristiques de l´échantillon:** un total de 101 patients était inclus parmi 126 survivants d´AVC éligibles durant la période du 1^er^ janvier 2012 au 31 juillet 2019, soit un taux de réponse de 79,5%. L´âge moyen des patients était de 57,2±10,5 ans avec des extrêmes de 29 et 80 ans. La sex-ratio était de 1,3. Environ 33,7% d´entre eux n´étaient pas scolarisés (Tableau 1). L´hypertension artérielle et le diabète étaient retrouvés chez respectivement 94,1% et 20,8% des patients (Tableau 1). La majorité des patients (92,1%) étaient sous traitement antihypertenseur. Plus de deux tiers (67,3%) d´entre eux avaient une pression artérielle élevée (pression artérielle systolique ≥ 140mmHg ou diastolique ≥ 90mmHg) le jour de la visite. L´hyperglycémie (glycémie capillaire à jeun ≥ 1,26g/l) était retrouvée chez 14,8% des patients le jour de la visite. Aucun patient ne consommait suffisamment de fruits et légumes. Parmi eux, 75,2% avaient une faible pratique d´activité physique; 8,9% étaient tabagiques et 11,9% des personnes obèses (Tableau 1). Des antécédents personnels (11,9%) et familiaux d´AVC (15,8%) étaient notés. Environ 57,4% des patients avaient fait un AVC ischémique (Tableau 1). Le délai médian depuis l´AVC était de 11 mois (intervalle interquartile: [2,0 à 23,5]) avec des extrêmes de 1 et 78 mois. Les patients ayant eu l´AVC depuis moins de 1 an étaient plus représentés (54,5%) (Tableau 1). Près de la moitié (48,5%) était sous des antiagrégants plaquettaires. Plus du dixième (13,9%) avait un handicap sévère (Tableau 1). A l´électrocardiogramme: 19,8% présentaient une déviation axiale gauche; 10,9% une tachycardie ou une bradycardie sinusale; 1,0% une fibrillation auriculaire; 18,8% des troubles conductifs; 24,8% une hypertrophie auriculaire gauche; et 37,6% une hypertrophie ventriculaire gauche.

**Tableau 1:** caractéristiques générales de la population étudiée, Parakou 2019

	Total	Hommes	Femmes
	Effectif (%)	Effectif (%)	Effectif (%)
**Age (ans)**			
<40	05 (04,9)	02 (03,6)	03 (06,7)
40-59	52 (51,5)	29 (51,8)	23 (51,1)
≥60	44 (43,6)	25 (44,6)	19 (42,2)
**Niveau d´instruction**			
Non scolarisé	34 (33,7)	09 (16,1)	25 (55,5)
Primaire	23 (22,8)	15 (26,8)	08 (17,8)
Secondaire et plus	44 (43,5)	32 (57,1)	12 (26,7)
**HTA**			
Non	06 (05,9)	04 (07,1)	02 (04,4)
Oui	95 (94,1)	52 (92,9)	43 (95,6)
**Diabète**			
Non	80 (79,2)	46 (82,1)	34 (75,6)
Oui	21 (20,8)	10 (17,9)	11 (24,4)
**Obésité**			
Non	89 (88,1)	51 (91,1)	38 (84,4)
Oui	12 (11,9)	05 (08,9)	07 (15,6)
**Inactivité physique**			
Non	25 (24,8)	17 (30,4)	08 (17,8)
Oui	76 (75,2)	39 (69,6)	37 (82,2)
**Statut tabagique**			
Non-fumeur	81 (80,2)	38 (67,8)	43 (95,6)
Ancien-fumeur	11 (10,9)	8 (14,3)	01 (02,2)
Fumeur	09 (08,9)	10 (17,9)	01 (02,2)
**Type AVC**			
Ischémique	58 (57,4)	31 (55,4)	27 (60,0)
Hémorragique	29 (28,7)	21 (37,5)	8 (17,8)
Indéterminé	14 (13,9)	04 (07,1)	10 (22,2)
**Délai AVC (ans)**			
< 1	55 (54,5)	31 (55,4)	24 (53,3)
[1-5]	41 (40,6)	23 (41,1)	18 (40,0)
≥5	05 (04,9)	02 (03,5)	03 (06,7)
**Rankin**			
≤1	40 (39,6)	22 (39,3)	18 (40,0)
[2-3]	47 (46,5)	29 (51,8)	18 (40,0)
≥4	14 (13,9)	05 (08,9)	09 (20,0)

AVC: accident vasculaire cérébral; HTA: hypertension artérielle; Inactivité physique: ≤ 150 minutes d´activité physique modérée ou équivalent par semaine

**Prévalence de la coronaropathie:** la prévalence hospitalière de la coronaropathie était estimée à 49,5% (intervalle de confiance 95% [39,4-59,6]). Cinq patients sur cent avaient un antécédent de coronaropathie confirmé. Près de six patients sur cent (5,8%) avaient une douleur thoracique suspecte d´angine de poitrine sur la base du questionnaire de dépistage et 44,6% des anomalies électrocardiographiques évocatrices de coronaropathie; ces anomalies étaient associées à une hypertrophie ventriculaire gauche dans 44,4% des cas. Le Tableau 2 présente la prévalence de la coronaropathie chez les survivants d´AVC suivant leurs caractéristiques générales.

**Tableau 2:** prévalence de la coronaropathie chez les survivants d´accident vasculaire cérébral en fonction de leurs caractéristiques générales, Parakou 2019

	Total	Coronaropathie (oui)	
		Effectif	%
**Sexe**			
Féminin	45	18	40,0
Masculin	56	32	57,1
**Age (ans)**			
<40	05	03	60,0
40-59	52	19	36,5
≥60	44	28	63,6
**Niveau d´instruction**			
Non scolarisé	34	18	52,9
Primaire	23	07	30,4
Secondaire et plus	44	25	56,8
**HTA**			
Non	06	05	83,3
Oui	95	45	47,4
**Diabète**			
Non	80	36	45,0
Oui	21	14	66,7
**Obésité**			
Non	89	41	46,1
Oui	12	09	75,0
**Inactivité physique**			
Non	25	10	40,0
Oui	76	40	52,6
**Statut tabagique**			
Non-fumeur	81	35	43,2
Ancien-fumeur	11	08	72,7
Fumeur	09	07	77,8
**Type AVC**			
Ischémique	58	28	48,3
Hémorragique	29	16	55,2
Indéterminé	14	06	42,9
**Délai AVC (ans)**			
< 1	55	27	49,1
[1-5[	41	22	53,7
≥5	05	01	20,0
**Rankin**			
≤1	40	21	52,5
[2-3]	47	22	46,8
≥4	14	07	50,0

AVC: accident vasculaire cérébral; HTA: hypertension artérielle; Inactivité physique: ≤ 150 minutes d´activité physique ou équivalent par semaine

**Facteurs associés à la coronaropathie:** en régression logistique uni-variée, aucun facteur n´avait une association statistiquement significative avec la coronaropathie au seuil de p inférieur à 0,05 (Tableau 3). Les facteurs associés à la coronaropathie en analyse multivariée étaient la non-scolarisation (p=0,036), l´obésité (p=0,036) et le tabagisme ancien (p=0,044) (Tableau 3). Les sujets ayant fait des études primaires avaient moins de risque d´avoir une coronaropathie (OR ajusté = 0,2; IC 95% [0,1-0,9]) comparativement aux sujets non instruits. Ceux qui présentaient une obésité étaient plus à risque de coronaropathie (OR ajusté = 5,0; IC 95% [1,1-22,3]) comparativement aux autres. Les anciens fumeurs avaient un risque plus élevé d´avoir une coronaropathie (OR ajusté = 5,6; IC 95% [1,1-30,4]) que les non-fumeurs.

**Tableau 3:** facteurs associés à la coronaropathie chez les survivants d´accident vasculaire cérébral, Parakou 2019

	Analyse uni-variée			Analyse multi-variée		
	ORb	IC 95%	p	ORa	IC 95%	p
**Sexe**						
Féminin	1			1		
Masculin	2,0	[0,9-4,4]	0,183	2,3	[0,8-6,6]	0,130
**Age (ans)**						
<40	1					
40-59	0,4	[0,1-2,5]	0,317			
≥60	1,2	[0,2-7,7]	0,873			
**Niveau d´instruction**						
Non scolarisé	1			1		
Primaire	0,4	[0,1-1,2]	0,097	0,2	[0,1-0,9]	**0,036**
Secondaire et plus	1,2	[0,5-2,9]	0,733	0,7	[0,2-2,4]	0,604
**HTA**						
Non	1			1		
Oui	0,2	[0,1-1,6]	0,124	0,3	[0,1-3,0]	0,289
**Diabète**						
Non	1			1		
Oui	2,4	[0,9-6,7]	0,082	2,4	[0,7-7,9]	0,160
**Obésité**						
Non	1			1		
Oui	3,5	[0,9-13,8]	0,073	5,0	[1,1-22,3]	**0,036**
**Inactivité physique**						
Non	1					
Oui	1,7	[0,7-4,2]	0,276			
**Statut tabagique**						
Non-fumeur	1			1		
Ancien-fumeur	3,5	[0,9-14,2]	0,079	5,6	[1,1-30,4]	**0,044**
Fumeur	4,6	[0,9-23,5]	0,067	2,6	[0,5-15,1]	0,288
**Type AVC**						
Ischémique	1					
Hémorragique	1,3	[0,5-3,2]	0,546			
Indéterminé	0,8	[0,2-2,6]	0,718			
**Délai AVC (ans)**						
< 1	1					
[1-5]	1,2	[0,5-2,7]	0,658			
≥5	0,3	[0,1-2,5]	0,241			
**Rankin**						
≤1	1					
[2-3]	0,8	[0,3-1,9]	0,597			
≥4	0,9	[0,3-3,1]	0,872			

AVC: accident vasculaire cérébral; HTA: hypertension artérielle; Inactivité physique: ≤ 150 minutes d´activité physique ou équivalent par semaine; ORb: odds ratio brut; ORa: odds ratio ajusté; IC: intervalle de confiance

## Discussion

Cette étude a porté sur des survivants d´AVC avec un âge moyen de 57 ans environ et une prédominance masculine. L´AVC était ischémique dans plus de la moitié des cas, avec comme principal facteur de risque l´hypertension artérielle. La coronaropathie était présente ou suspectée chez près de la moitié de l´échantillon. Dans la littérature, la prévalence de la coronaropathie varie considérablement en fonction de la méthodologie, notamment en fonction des outils de diagnostic (électrocardiogramme de repos, électrocardiogramme d´effort, échographie d´effort, scintigraphie myocardique, coroscanner, coronarographie), et de la population d´étude. La prévalence de la coronaropathie estimée dans cette étude est semblable à celle retrouvée chez des survivants d´AVC par Sowanou *et al*. en 2013 à Parakou au Bénin (47,4%), sur la base des antécédents, des données cliniques et électrocardiographiques [9]. Une prévalence similaire (52%) avait été notée en Espagne dans une étude réalisée par Arenillas *et al*. en 2005 [14]. Des prévalences plus élevées avaient été rapportées par Love *et al*. en 1992 aux Etas Unis (58%) et Kovacik *et al*. en Slovaquie en 2012 (71,8%) [15,16]. Par contre, des prévalences plus faibles entre 15 et 40% ont été notées en Tanzanie (Afrique), en Suisse, au Japon et en France [2,17-21]. Dans l´étude tanzanienne qui a utilisé l´électrocardiographie comme moyen de dépistage, la faible prévalence retrouvée (23,7%) pourrait s´expliquer par le fait que les séquelles d´infarctus du myocarde étaient le seul critère utilisé pour détecter la coronaropathie [2].

L´âge n´était pas significativement associé à la coronaropathie. Néanmoins, la prévalence était plus élevée chez les sujets d´âge supérieur à 60 ans (63,6%) comparativement aux autres. Ce résultat confirme l´impact de l´âge sur le développement de maladie cardiovasculaire. Le vieillissement de la paroi des artères avec l´âge favorise une certaine prédisposition à la maladie athéromateuse surtout en présence des autres facteurs de risque cardiovasculaire [22]. Une étude sur un échantillon plus grand pourrait faire ressortir sur le plan statistique le lien entre l´âge et la présence de coronaropathie chez les survivants d´AVC. L´association entre le sexe et la coronaropathie n´a pas été retrouvée. Mais certains auteurs ont noté un lien statistiquement significatif entre le sexe masculin et la coronaropathie [20,23]. Le niveau d´instruction était associé à la coronaropathie (p=0,036). Le fait que les patients non scolarisés aient un risque plus élevé de coronaropathie peut s´expliquer par une exposition plus fréquente aux facteurs de risque par manque d´information. L´obésité et le tabagisme ancien étaient également associés à la coronaropathie. Ces associations sont logiquement attendues car l´obésité et le tabagisme sont des facteurs de risque cardiovasculaire prouvés dans la littérature. Une étude plus large pourrait confirmer l´association entre le tabagisme actuel et la présence de coronaropathie chez les survivants d´AVC, rapportée par certains auteurs [20,24].

Les autres facteurs de risque cardiovasculaire majeurs comme l´HTA et le diabète n´étaient pas associés à la coronaropathie dans notre étude. Toutefois, leur association avec la coronaropathie chez les survivants d´AVC a été observée dans d´autres études [20,25,26]. Cette étude a utilisé des outils simples et accessibles de dépistage d´anomalies évocatrices de coronaropathie utilisables en milieu de soins dépourvu de plateau technique adéquat (revue de dossier médical, questionnaire de l´OMS pour le dépistage de l´angine de la poitrine, ECG de repos). Le code Minnesota utilisé pour la lecture des enregistrements électrocardiographiques confère un cadre consensuel validé pour l´interprétation des tracés. Le questionnaire de l´Organisation mondiale de la santé est reconnu pour le dépistage de l´angor stable dans les études épidémiologiques. Néanmoins, la prévalence de la coronaropathie a pu être surestimée par les moyens diagnostiques utilisés. En effet, les anomalies de la repolarisation peuvent être induites par l´hypertrophie ventriculaire gauche présente chez plus du tiers des patients de notre série. L´étude comble l´insuffisance de données sur la présence d´anomalies suspectes de coronaropathie chez les patients survivants d´AVC. Elle montre l´intérêt de la réalisation d´un bilan de recherche de la diffusion de la maladie athéromateuse chez les survivants d´AVC en particulier dans les cas d´AVC ischémique.

## Conclusion

La fréquence de la coronaropathie est élevée chez les patients victimes d´AVC au-delà de la phase aiguë. Certains facteurs de risque expliqueraient la présence de la coronaropathie notamment la non-scolarisation, l´obésité et le tabagisme ancien. Dans le suivi des survivants d´AVC, il est important de rechercher de manière systématique la coronaropathie afin d´optimiser la prise en charge.

### Etat des connaissances sur le sujet

- La coronaropathie et les accidents vasculaires cérébraux ischémiques partagent la même étiologie principale (athérosclérose) et donc les mêmes facteurs de risque. La coronaropathie et les accidents vasculaires cérébraux peuvent coexister et aggraver le pronostic vital des patients à court, à moyen et long terme;
- Il existe des outils diagnostiques invasifs et non invasifs de performance connue pour dépister les coronaropathies chez les patients à haut risque.

### Contribution de notre étude à la connaissance

- Les études sur la fréquence des coronaropathies chez les survivants d´AVC sont rares en Afrique subsaharienne;
- La coronaropathie a été définie à partir de critères simples. Les données montrent la fréquence élevée des anomalies évocatrices de coronaropathies chez les survivants d´AVC, soulignant ainsi l´intérêt de leur détection pour l´optimisation de la prise en charge.
